# Biomarkers as predictors of mortality in critically ill obese patients with COVID-19 at high altitude

**DOI:** 10.1186/s12890-023-02399-3

**Published:** 2023-04-06

**Authors:** Jorge Luis Vélez-Páez, Santiago Xavier Aguayo-Moscoso, Christian Castro-Bustamante, Mario Montalvo-Villagómez, Fernando Jara-González, Lucy Baldeón-Rojas, Natalia Zubieta-DeUrioste, Denise Battaglini, Gustavo R. Zubieta-Calleja

**Affiliations:** 1 Centro de Investigación Clínica, Hospital Pablo Arturo Suárez, Unidad de Terapia Intensiva, Quito, Ecuador; 2 Facultad de Ciencias Médicas, Universidad Central del Ecuador, Quito, Ecuador; 3 Instituto de Investigación en Biomedicina, Universidad Central del Ecuador, Quito, Ecuador; 4 High Altitude Pulmonary & Pathology Institute (HAPPI-IPPA), La Paz, Bolivia; 5 IRCCS Ospedale Policlinico San Martino, Genova, Italy

**Keywords:** Altitude, Obese, COVID-19, Coronavirus disease, Obesity

## Abstract

**Background:**

Obesity is a common chronic comorbidity of patients with COVID-19, that has been associated with disease severity and mortality. COVID-19 at high altitude seems to be associated with increased rate of ICU discharge and hospital survival than at sea-level, despite higher immune levels and inflammation. The primary aim of this study was to investigate the survival rate of critically ill obese patients with COVID-19 at altitude in comparison with overweight and normal patients. Secondary aims were to assess the predictive factors for mortality, characteristics of mechanical ventilation setting, extubation rates, and analytical parameters.

**Methods:**

This is a retrospective cohort study in critically ill patients with COVID-19 admitted to a hospital in Quito-Ecuador (2,850 m) from Apr 1, 2020, to Nov 1, 2021. Patients were cathegorized as normal weight, overweight, and obese, according to body mass index [BMI]).

**Results:**

In the final analysis 340 patients were included, of whom 154 (45%) were obese, of these 35 (22.7%) were hypertensive and 25 (16.2%) were diabetic. Mortality in obese patients (31%) was lower than in the normal weight (48%) and overweight (40%) groups, but not statistically significant (*p* = 0.076). At multivariable analysis, in the overall population, older age (> 50 years) was independent risk factor for mortality (B = 0.93, Wald = 14.94, OR = 2.54 95%CI = 1.58–4.07, *p* < 0.001). Ferritin and the neutrophil/lymphocyte ratio were independent predictors of mortality in obese patients. Overweight and obese patients required more positive and-expiratory pressure compared to normal-weight patients. In obese patients, plateau pressure and mechanical power were significantly higher, whereas extubation failure was lower as compared to overweight and normal weight.

**Conclusions:**

This preliminary study suggests that BMI was not associated with mortality in critically ill patients at high altitude. Age was associated with an increase in mortality independent of the BMI. Biomarkers such as ferritin and neutrophils/lymphocytes ratio were independent predictors of mortality in obese patients with COVID-19 at high altitude.

## Background

The coronavirus disease 2019 (COVID-19) pandemic, as a major health crisis, led to an incredible scientific effort to understand how to properly manage patients with COVID-19 [1]. The literature revealed that increasing age and patient comorbidities are associated with more severe diseases and adverse outcomes [2]. The most common chronic comorbidity in COVID-19 patients was obesity, which was rather associated with severe illness and death instead of more intensive care unit (ICU) admissions [3]. Obesity, per se*,* is related to an increased prevalence of other diseases such as renal insufficiency, cardiovascular diseases, diabetes mellitus, endothelial dysfunction, and hypertension. These conditions have been identified as major risk factors for disease severity and mortality in COVID-19 [4–6].

The impact of high altitude on COVID-19 outcome has been poorly investigated. Initial observations showed that mortality was lower at higher altitude [7]. Patients at high altitude were more likely to experience ICU discharge and hospital survival than those treated at sea-level [8–11]. Rodriguez et al. in more than 5,000 patients with COVID-19 at more than 2,500 m of altitude, showed that a higher body mass index (BMI) was associated with greater survival, regardless of severity. Older age, low arterial partial pressure of oxygen (PaO_2_)/ fraction of inspired oxygen (FiO_2_), and high lactate dehydrogenase (LDH) during admissions were predictors of mortality in this cohort [12]. Moreover, patients with COVID-19 at high altitude seem to experience increased inflammation as compared with those at sea level [13]. Immune levels at high altitude have been found elevated as compared with sea level [14]. Possible explanations for these findings are genetic and physiological adaptations due to exposure to chronic hypoxia [13, 15–17]. The association between obesity and altitude in patients with COVID-19 has not been investigated yet. Initial data indicate that diabetes and obesity are linked to greater mortality rates, but little research has been done in relation to altitude [18].

The primary aim of this study was to investigate the survival rate of critically ill obese patients with COVID-19 at altitude (2,850 m) in comparison with overweight and normal weight patients. Secondary aims were to assess predictive factors for mortality, characteristics of mechanical ventilation setting, extubation rates, and analytical parameters.

## Methods

### Study design

This retrospective cohort study was performed at the Pablo Arturo Suárez General Provincial Hospital, located in Quito, Ecuador at 2,850 m, which is a COVID-19 exclusive care center for symptomatic respiratory patients who require hospitalization. All patients hospitalized in the intensive care unit (ICU) from Apr 1, 2020, to Nov 1, 2021, with a positive polymerase chain reaction test (RT-PCR) or with a positive nasal swab upon admission to the emergency department or hospitalization referred to the Pablo Arturo Suárez General Provincial Hospital, Quito, Ecuador, were included. The study was approved by the Human Research Ethics Committee of the Calderón Hospital (number of approval MSP-CZ9HGDC-2022–0914-O). Informed consent was waived due to the retrospective nature of this study in accordance with local regulations. All experiments were performed in accordance with relevant guidelines and regulations and with the Declaration of Helsinki.

### Inclusion and exclusion criteria

All patients older than 18 years, who required invasive mechanical ventilation and with a confirmed diagnosis of COVID-19 (vaccines were not yet available) were included.

### Data collection

Data were collected by physicians trained in critical care, anonymized, and stored as electronic clinical records at the Pablo Arturo Suárez General Provincial Hospital, Quito-Ecuador. Data collection included clinical-epidemiological variables such as age, sex, co-morbidities (diabetes mellitus, arterial hypertension), clinical scales such as the Sequential Organ Failure Assessment (SOFA) and Acute Physiology and Chronic Health Evaluation (APACHE II), days of hospitalization, mortality at 28 days, as well as the following analytical variables: D-dimer (Normal Values [NV]: 0.0–500 ng/ml), ferritin (NV: 22–322 ng/ml), lactic dehydrogenase (LDH) (NV: 135 -214 U/L), interleukin-6 (IL-6) (NV: 0.0–3.4 pg/ml), neutrophil–lymphocyte ratio (NLR), and absolute blood lymphocyte count (NV: 1000 to 3200/microLiter); and variables related to lung mechanics such as positive end-expiratory pressure (PEEP), static compliance of the respiratory system (Crs), driving pressure (⊗ P), mechanical power (MP), and average tidal volume (VT). These variables were obtained upon admission to the ICU and later at around 48 and 72 h. Gas exchange was collected, including PaO_2_/FiO_2_, pH, bicarbonate, arterial partial pressure of carbon dioxide (PaCO_2_). The hemoglobin, hematocrit, absolute lymphocyte count, and the NLR were obtained from the routinary blood count, which was measured using an automated hematology analyzer (Advia 2120i, USA). Ferritin and IL-6 were evaluated by chemiluminescence (Inmulite 2000 XPi, USA). LDH was measured by photometry (Advia 1800), and D-dimer by fluorescence enzyme-linked immunosorbent assay (ELISA). The measurement of the ventilatory mechanics was performed in quasi-static flow, on patients who were sedated and with muscle relaxants placed under a controlled mechanical ventilator strategy.

### Definitions

Study participants were classified based on their body mass index (BMI) as normal (BMI: 18.5 to 24.9 kg/m^2^), overweight (BMI: 25 to 29.9 kg/m^2^), and obese (BMI: > 30 kg/m^2^). BMI was calculated according to the standard formula: weight (kg) / height^2^ (m) [19]. When classifying for sub-groups of obesity, obesity type I was defined as BMI of 30 to < 35, type II as BMI of 35 to < 40, type III as BMI of 40 or higher [20].

The following formulas were used to calculate respiratory parameters [21]: mechanical power in pressure control ventilation (PCV): $\mathrm{MPPCV}= 0.098 \times VT \times (P +PEEP)\times RR$. Static respiratory system compliance: Crs = ΔV/ ΔP L/cmH_2_O.

### Statistical analysis

Data are reported as median and interquartile ranges [IQR = 25th − 75th percentiles], mean (standard deviation, SD), or numbers (percentages), as appropriate. A bivariate analysis was performed to compare clinical characteristics and laboratory parameters between normal weight, overweight, and obese patients. Categorical variables were evaluated with the chi-square test. Quantitative variables were assessed with parametric distribution, the ANOVA test (Bonferroni test for post-hoc comparison) and the Kruskal Wallis test (Bonferroni test for post-hoc comparison) in non-parametric distribution.

At univariate analysis, the predictor candidates to enter the multivariate logistic regression analysis were considered for *p* < 0.05. Age, ferritin, D-dimer, NLR, LDH, SOFA, and APACHE were included as possible predictors. Multivariate logistic regression analysis stratified by BMI classification was performed to determine mortality predictors.

Multivariate logistic regression analysis to determine mortality predictors was stratified by BMI classification and adjusted by age (the variable was dichotomized into > 50 and < 50 years based on the median age of the general population).

Statistical significance for comparing proportions and means was established at *p* < 0.05. The Odds Ratio (OR) was considered significant by observing the 95% confidence interval (CI) limits, where a risk factor was considered if the lower limit > 1. All statistical analyses were performed using the R® software (version 4.1.2).

## Results

### Demographic characteristics of the overall population and according to BMI groups

**Fig. 1:**
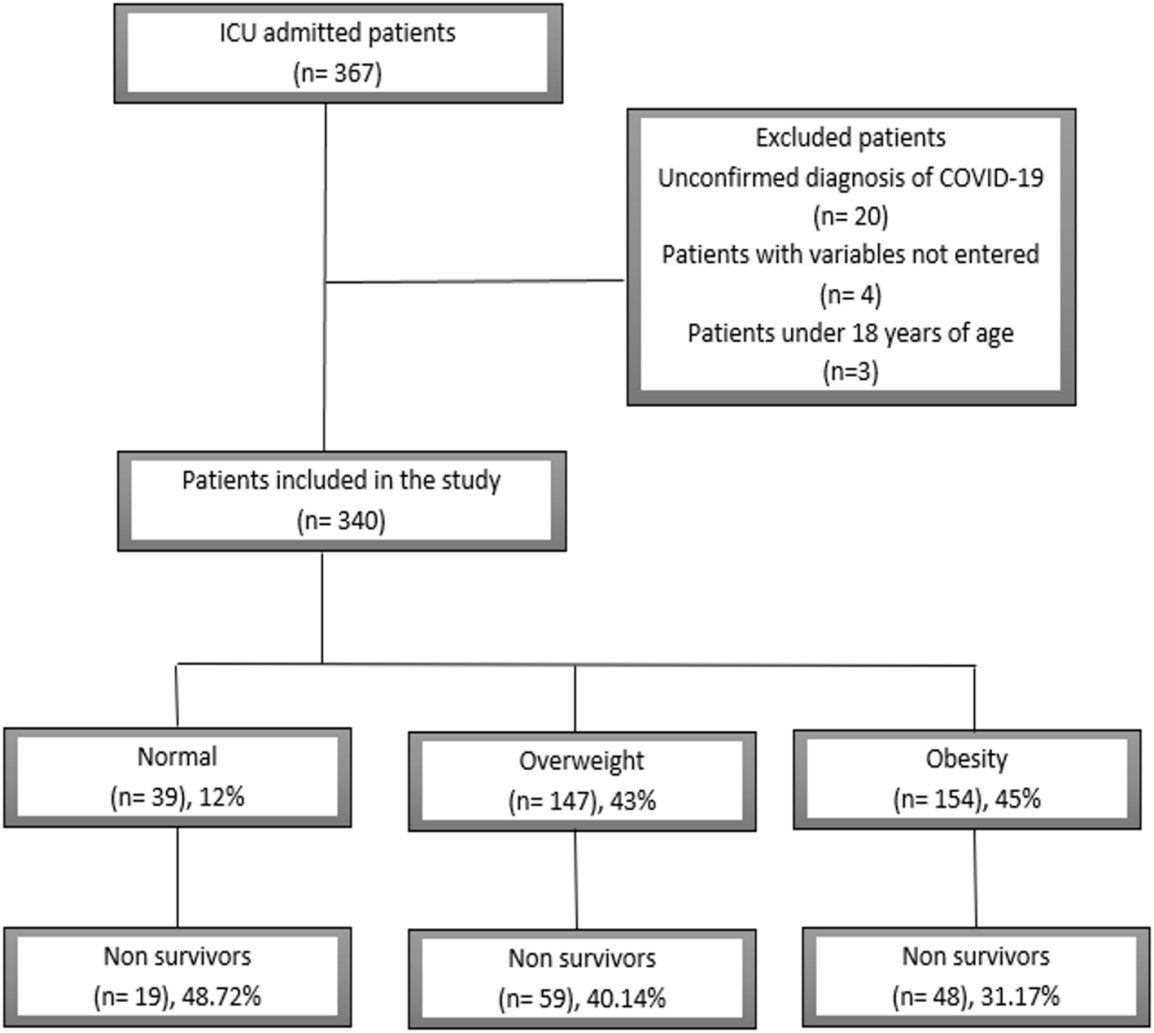
Flow chart of patient inclusion during the study timeframe

From the 367 patients screened for inclusion, 340 patients with COVID-19 were analyzed (Fig. 1). Our cohort was composed by 12% (*n* = 39) normal weight patients, 43% (*n* = 147) overweight patients, and 45% (*n* = 154) obese patients. Average age of overall cohort was 50.84 (standard deviation 13.14) years [54.95 (15.83) years in normal weight, 52.37 (12.24) years in overweight, and 48.35 (12.82) years in obese groups, *p* = 0.003]. Most of the patients, were males (72.06% *n* = 245). Overweight patient were more frequently males, whereas obese patients were more frequently females (*p* = 0.046). Arterial hypertension was more common in the obesity group in comparison with the overweight and normal weight groups, *p* = 0.018. Table 1 presents the clinical characteristics of the overall population and according to BMI classification.

**Table 1:** Clinical characteristics of patients in the overall population and according to BMI classification

Clinical Characteristics	Total (*n* = 340)	BMI Classification			*p*-value
		**Normal** **(*n* = 39)**	**Overweight** **(*n* = 147)**	**Obesity** **(*n* = 154)**	
Age (mean (SD)) years ^1/^	50.84 (13.14)	54.95 (15.83)	52.37 (12.24)	48.35 (12.82)	0.003^*^
Gender (n (%)) ^2/^					
Male	245 (72.06)	27 (11.02)	116 (47.35)	102 (41.63)	0.046^*^
Female	95 (27.94)	12 (12.63)	31 (32.63)	52 (55.91)	
DM (n (%)) ^2/^	45 (13.24)	3 (7.69)	17 (11.56)	25 (16.23)	0.272
HTA (n (%)) ^2/^	56 (16.47)	4 (10.26)	17 (11.56)	35 (22.73)	0.018^*^
Apache II at admission (median (IQR)) ^3/^	16 (11–20.75)	16 (11.25–20)	15 (11–20)	17 (12–21)	0.796
SOFA (median (IQR)) ^3/^					
24 h	7 (4–9)	6.5 (5–9)	6 (4–8)	7 (4–10)	0.104
48 h	5 (3–7)	6 (3–8.75)	5 (3–7)	6 (4–8)	0.051
72 h	4 (3–7)	5 (3–9)	4 (3–7)	4 (3–7)	0.652
Hospitalization days (median (IQR)) ^3/^	9 (6–15)	10 (6–16)	9.5 (6–15)	9 (6–15)	0.940
Mortality (n (%))^2/^	126 (37.06)	19 (48.72)	59 (40.14)	48 (31.17)	0.076

*DM* diabetes mellitus, *HTA* arterial hypertension, *SOFA* Sequential Organ Failure Assessment, *SD* Standard Deviation, *IQR* Interquartile Range

^*^significant differences; 1/ ANOVA (Bonferroni post-test); 2/ Chi-square test; 3/Kruskal Wallis test (Bonferroni post-test); different superscripts indicate weight classification group that differ in relation to clinical characteristics

### Mortality rate and predictors in the overall population and according to BMI groups

Mortality rate was 37.06% in the overall population, 48.72% in normal weight, 40.14% in overweight, and 31.17% in obese groups respectively, without significant differences among BMI groups, *p* = 0.076 (see Fig. 1).

At multivariable analysis, in the overall population, older age (> 50 years) was independent risk factor for mortality (B = 0.93, Wald = 14.94, OR = 2.54 95%CI = 1.58–4.07, *p* < 0.001). Obesity and overweight were not predictors for mortality (obesity: B = -0.55, Wald = 2.16, OR = 0.58, 95%CI 0.28–1.20, *p* = 0.14; overweight: B = -0.29, Wald = 0.612, OR = 0.75, 95%CI = 0.36–1.55, *p* = 0.43).

In the normal weight group, at 24 h, increased ferritin was identified as independent predictor for mortality (*p* = 0.043). For each ng/dl ferritin increase, the risk of mortality increased by 1% (Table 2).

**Table 2:** Multivariate regression to determine prognostic factors for mortality in COVID-19 patients stratified by BMI classification

Variables	B	Wald	*p*-value	OR (CI 95%)
**Normal weight**				
Age	0.52	0.28	0.599	1.68 (0.24–11.73)
Ferritin 24 h	0.00	4.10	0.043^*^	1.01 (1.00–1.02)
SOFA 24 h	0.42	2.50	0.114	1.53 (0.90–2.57)
APACHE 24 h	-0.02	0.02	0.878	0.98 (0.74–1.29)
NLR 24 h	0.02	0.71	0.400	1.02 (0.97–1.07)
D-Dimer 24 h	0.00	0.05	0.823	1.00 (1.00–1.00)
LDH 24 h	0.00	0.40	0.527	1.00 (1.00–1.00)
**Overweight**				
Age	0.87	4.55	0.033^*^	2.39^a^ (1.07–5.33)
Ferritin 24 h	0.00	0.00	0.993	1.00 (1.00–1.00)
SOFA 24 h	0.04	0.31	0.575	1.04 (0.90–1.21)
APACHE 24 h	0.04	1.32	0.250	1.04 (0.97–1.12)
NLR 24 h	0.01	1.20	0.272	1.01 (0.99–1.03)
D-Dimer 24 h	0.00	0.48	0.490	1.00 (1.00–1.00)
LDH 24 h	0.00	2.37	0.124	1.00 (1.00–1.00)
**Obese**				
Age	0.38	0.83	0.363	1.46 (0.65–3.30)
Ferritin 24 h	0.00	6.04	0.014^*^	1.03^*^ (1.01–1.04)
SOFA 24 h	0.02	0.08	0.771	1.02 (0.87–1.21)
APACHE 24 h	0.02	0.15	0.697	1.02 (0.94–1.10)
NLR 24 h	0.06	12.20	< 0.001^*^	1.06^*^ (1.03–1.10)
D-Dimer 24 h	0.00	3.54	0.060	1.00 (1.00–1.00)
LDH 24 h	0.00	3.45	0.063	1.00 (1.00–1.00)

*NLR* Neutrophils to lymphocytes ratio, *LDH* lactate dehydrogenase

^*^significant variable *p*-value < 0.05

^a^OR = significant odds ratio; based on logistic regression

In the overweight group, age was an independent predictor for mortality (*p* = 0.033), where for each year of increase in age the risk of mortality is 2.39 times greater (Table 2).

In the obese group, ferritin and NLR were independent predictors for mortality, *p* = 0.014 and *p* =  < 0.001 respectively. For each ng/dl increase in ferritin, the risk for mortality increased by 3%, whereas, for each unit increase in NLR, the risk for mortality increased by 6% (Table 2).

### Characteristics of mechanical ventilation setting, gas exchange, and extubation according to BMI groups

Tidal volume (VT) did not differ among groups nor over time. Positive end-expiratory pressure (PEEP) at 24, 48, and 72 h was higher in the obese and overweight groups when compared with the normal weight group, *p* ≤ 0.001, Table 3. Plateau pressure at 24 and 48 h was higher in the overweight and obese group in comparison with normal weight group (at 24 h *p* = 0.033, and at 48 h *p* = 0.015). Compliance and driving pressure did not differ among groups nor over time. Mechanical power at 24 and 48 h was higher in the overweight and obese groups in comparison with normal weight group. (at 24 h *p* = 0.004, at 48 h *p* = 0.024). No differences in gas exchange, PaO_2_/FiO_2_, nor PaCO_2_, were identified among groups nor across time.

**Table 3:** Mechanical ventilation parameters, gas exchange and extubation according to BMI groups

Mechanical ventilation parameters	Classification BMI			*p*-value
	**Normal** **(*n* = 39)**	**Overweight** **(*n* = 147)**	**Obesity** **(*n* = 154)**	
Admission ventilation mode (n (%)) ^1/^				
Volume controlled	1 (2,63)	10 (6,9)	6 (4)	0,403
Pressure controlled	37 (97.37)	135 (93.1)	144 (96)	
VT 24 h (median (IQR)) ^2/^ ml/kg of PBW	6.28 (6–7.3)	6.85 (6–7.5)	6.9 (6–7.7)	0.286
VT 48 h (median (IQR)) ^2/^ ml/kg of PBW	6.89 (6–7.3)	7 (6–8)	7 (6.2–7.8)	0.972
VT 72 h (median (IQR)) ^2/^ ml/kg of PBW	6.65 (6–7.88)	7 (6.1–7.93)	7 (6.2–7.8)	0.770
PEEP 24 h (median (IQR)) ^2/^ cmH_2_O	10 (8-10)	11 (9.75–12)	11 (8–12)	0.001^*^
PEEP 48 h (median (IQR)) ^2/^ cmH_2_O	8 (7–9)	9 (8–10)	9 (8–10)	< 0.001^*^
PEEP 72 h (median (IQR)) ^2/^ cmH_2_O	7 (5.25–8)	8 (6–9)	8 (7–10)	0.001^*^
Plateau pressure 24 h (median (IQR)) ^2/^ cmH_2_0	21 (19.25–25)	23 (21–25)	23 (22–26)	0.033^*^
Plateau pressure 48 h (median (IQR)) ^2/^ cmH_2_0	21 (18–23)	22 (20–24)	22 (20–25)	0.015^*^
Plateau pressure 72 h (median (IQR)) ^2/^ cmH_2_0	19.5 (15.25–22)	21 (19–23)	21 (19–24)	0.075
Compliance 24 h (median (IQR)) ^2/^ ml/ cmH_2_0	35 (29–43)	35 (27.75–40)	32.5 (24.75–40)	0.576
Compliance 48 h (median (IQR)) ^2/^ ml/ cmH_2_0	38 (27–45)	35 (30–42.25)	33 (25–40.25)	0.149
Compliance 72 h (median (IQR)) ^2/^ ml/ cmH_2_0	34 (27.33–46.5)	36 (32–42)	34 (29–42)	0.130
Driving pressure 24 h (median (IQR)) ^2/^ cmH_2_0	12 (10–14.75)	13 (11–14)	13 (12–15)	0.429
Driving pressure 48 h (median (IQR)) ^2/^ cmH_2_0	12 (10.25–14)	13 (12–15)	13 (12–15)	0.458
Driving pressure 72 h (median (IQR)) ^2/^ cmH_2_0	12.5 (10–14.75)	13 (12–14)	13 (11–15)	0.460
Mechanical power 24 h (median (IQR)) ^2/^ j/min	13.6 (11.25–16)	16 (14–18)	16 (14–18.4)	0.004^*^
Mechanical power 48 h (median (IQR)) ^2/^ j/min	13.5 (12–14.78)	15 (13.7–17)	16 (14–19)	0.024^*^
Mechanical power/Crs 24 h (median (IQR)) ^2/^ j/min	0.39 (0.30–0.58)	0.47 (0.38–0.61)	0.49 (0.39–0.71)	0.031^*^
Mechanical power/Crs 48 h (median (IQR)) ^2/^ j/min	0.39 (0.30–0.49)	0.42 (0.33–0.53)	0.48 (0.36–0.70)	0.009^*^
PaCO_2_ 24 h (median (IQR)) ^2/^ mmHg	39.5 (32.25–44.75)	42 (38–49.25)	42 (38–50)	0.057
PaCO_2_ 48 h (median (IQR)) ^2/^ mmHg	42 (36–51.5)	42 (36.75–47.25)	42 (37–50)	0.489
PaCO_2_ maximum 72 h (median (IQR)) ^2/^ mmHg	41 (36.5–43.75)	41 (37–47)	44 (39–48)	0.471
PaO_2_/FiO_2_ 24 h (median (IQR)) ^2/^ mmHg	166 (118.5–190.75)	152.5 (108.75–172.75)	140 (105–165)	0.057
PaO_2_/FiO_2_ 48 h (median (IQR)) ^2/^ mmHg	164 (131.25–195.5)	170 (148–188)	162 (132–180)	0.298
PaO_2_/FiO_2_ 72 h (median (IQR)) ^2/^ mmHg	161 (139.38–189.25)	166.5 (139.25–196.25)	167 (140–197)	0.730
Prone ventilation (n (%)) ^1/^	16 (41.03)	88 (59.86)	92 (59.74)	0.083
Pronation days (median (IQR)) ^2/^	2 (0–2.75)	2 (0–3)	2 (0–3)	0.357
Use of neuromuscular blocker (n (%)) ^1/^	17 (44.74)	84 (57.53)	94 (61.44)	0.174
Days with neuromuscular blocker (median (IQR)) ^2/^	2 (0–3)	2 (0–2)	2 (0–2)	0.643
Days on IMV (median (IQR)) ^2/^	8 (7–14.5)	7 (4–13)	8 (5–13)	0.282
Extubation (n (%)) ^1/^				
Failed	12 (46.15)	27. (27.00)	22 (18.97)	0.013^*^
Successful	14 (53.85)	73. (73.00)	94 (81.03)	

*IQR* Interquartile Range, *PBW* Predicted body weight, *PaCO*_*2*_ Arterial partial pressure of carbon dioxide, *VT* Tidal volume, *PEEP* Positive end-expiratory pressure, *PaO*_*2*_ Arterial partial pressure of oxygen, *FiO*_*2*_ Fraction of inspired oxygen, *IMV* invasive mechanical ventilation

^*^significant differences; 1/ Chi-square test; 2/ Kruskal Wallis test (Bonferroni post-test); different superscripts indicate weight classification group that differ in relation to clinical characteristics

Extubation was significantly more successful in the obese group (81.03%) in comparison with the normal weight group (53.85%), *p* = 0.013.

### Analytical parameters

The comparison of analytical parameters at 24 h among different BMI groups is reported in Table 4.

**Table 4:** Analytical parameters according to BMI groups

Analytics Parameters	Classification BMI			*p*-value
	**Normal** **(*n* = 39)**	**Overweight** **(*n* = 147)**	**Obesity** **(*n* = 154)**	
D-dimer 24 h (median (IQR)) ng/ml	1204 (779.73–1932.75)	1113 (719.5–2854.5)	977.5 (669.65–1894)	0.116
Ferritin 24 h (median (IQR)) ng/ml	1259.8 (657–1650)	1326.5 (705.18–1650)	1064.45 (644.6–1500)	0.034^*^
LDH 24 h (median (IQR)) U/L	746 (547–1001)	804 (645.5–992)	783.5 (579.5–1047.25)	0.483
IL-6 (median (IQR)) pg/mL	34.75 (17.23–120.25)	27 (12.15–80.83)	27.2 (12.78–60.53)	0.393
Lymphocytes 24 h (median (IQR)))	630 (400–1020)	550 (360–860)	630 (445–895)	0.071
NLR 24 h (median (IQR))	13 (8.2–22.56)	17.58 (9.92–29.34)	14.29 (9.14–20.83)	0.052
Hemoglobin (median (IQR))	15.2 (13.8–16.6)	16 (14.9–17.2)	16.2 (14.78–17.23)	0.112
Hematocrit (median (IQR))	45.3 (41.5–48.3)	47.4 (44.2–51.3)	48.05 (43.98–51.9)	0.071

*IQR* Interquartile Range, *NLR* neutrophils to lymphocytes ratio, *LDH* lactate dehydrogenase

^*^significant differences. Kruskal Wallis test (Bonferroni post-test)

**Fig. 2:**
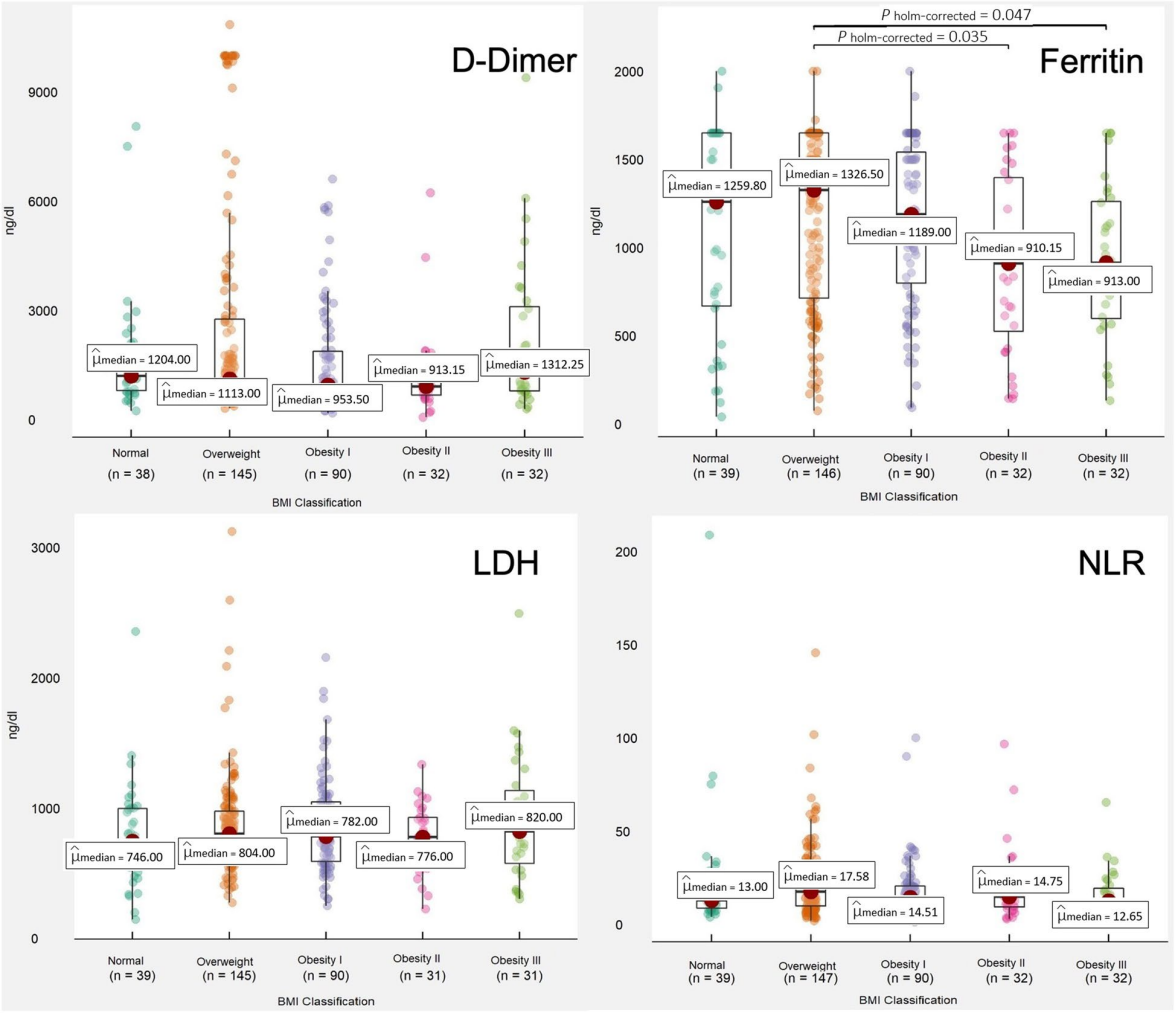
Comparison of analytical parameters according to BMI classification. The obesity group is divided into three sub-groups (obesity type I, type II, and type III). At 24 h, no differences were found among BMI groups for D-Dimer (*p* = 0.097), LDH (*p* = 0.785), and NLR (*p* = 0.155). Ferritin values significantly differed among groups (*p* = 0.007). *NLR* neutrophils to lymphocytes ratio, *LDH* lactate dehydrogenase

At 24 h, we did not find significant differences in D-dimer concentrations among groups (Fig. 2). Ferritin significantly differed among BMI groups, p = 0.034, being ferritin values in the normal weight, overweight, and obesity groups of 1259.8 ng/ml, 1326.5 ng/ml, and 1064.45 ng/ml, respectively (Table 4). Additionally, we found differences in ferritin levels among overweight (1326.50) ng/dl, type II obesity (910.15) ng/dl, and type III obesity (913 ng/dl), *p* = 0.007 (Fig. 2). LDH and NLR did not significantly differed according to the BMI classification (Fig. 2).

## Discussion

To the best of our knowledge, this is one of the first studies investigating possible predictors of mortality in obese patients with COVID-19 living at high altitude. The main findings of our study can be summarized as follow: (1) the rate of mortality was 48.72% in patients with normal weight, 40.14% in patients with overweight, and 31.17% in obese patients, without significant differences among groups; (2) age > 50 years was an independent predictor of mortality in the overall population; at 24 h, ferritin was a predictor for mortality in the normal weight group, age in the overweight group, whereas ferritin and NLR in the obese group; (3) VT, compliance, and driving pressure did not differ among groups and over time, whereas PEEP levels, Plateau pressure, and mechanical power were higher in obese in relation to normal weight and overweight patients. No differences in gas exchange were found; (4) extubation was more successful in the obese group in comparison with normal weight and overweight groups, (5) at 24 h, ferritin was significantly higher in the overweight group followed by the normal and obesity groups respectively.

### Mortality and predictors for mortality

COVID-19 has shaken the world with severe aggressiveness in the lungs that we refer to as “pneumolysis” [22, 23], also affecting several organs [24, 25]. Some reports suggested that COVID-19 would have a catastrophic effect in high-altitude cities due to life conditions under chronic hypoxia. Nevertheless, previous experience gave us a different outlook [26–29]. At high altitude, it seems that the incidence of COVID-19 is lower, as well as the case fatality rate [16, 30–32], suggesting that chronic hypoxia should be considered a protective factor in COVID-19. In our study, the rate of mortality was 48.72% in patients with normal weight, 40.14% in patients with overweight, and 31.17% in obese patients, without differences among groups. This is in contrast with a previous finding in which obese patients (BMI > 30 kg/m^2^) with COVID-19 had 5 times higher risk of mortality than normal weight subjects. However, this study was conducted at sea level, and this may have influenced the results [33], as well as by the fact that our obese patients were younger than normal weight and overweight patients. Indeed, our study showed that in Quito, Ecuador, at high altitude (2,850 m), age > 50 years is an independent predictor of mortality in COVID-19 patients. The obesity paradox exists and has been reported in important studies with a larger number of patients [34]. In our study, the small sample of patients with normal weight compared to overweight and obesity could have biased the mean age, without showing the real effect of obesity as a protective factor at high altitude, as reported by some literature. Due to the scarcity of investigation on this topic, other confounding factors were searched, without finding a clear explanation [35, 36]. However, some observations at sea level found that several patient co-morbidities increased the fatal outcomes [37], and obesity was considered as an essential aggravating factor [30, 38, 39].

In our study, we investigated if there was any association between altitude, obesity, and COVID-19 and biomarkers as risk factors for mortality. We found no differences in D-dimer, LDH, IL-6 at 24 h according with the BMI classification. However, we found that ferritin was independent risk factor for mortality both in normal weight and obese patients. This association has been previously investigated by Mehanna et al. where increased C-reactive protein, serum ferritin, D-dimer, and LDH were independent risk factors for mortality in obese patients as compared with overweight and normal weight patients [40]. These results highlight a significant consequence of obesity in terms of the severity of the inflammatory response in obese patients with COVID-19.

Regarding white blood cell count, Ballaz et al. [41] did not find significant differences between the non-severe and severe groups. Excess neutrophils in severe cases are likely to cause a compensatory decrease in lymphocytes during the progression to severe COVID-19. NLR, a biomarker of systemic inflammatory response, predicted admission to the ICU in severe patients [41–44]. In our study, NLR was an independent predictor of mortality in obese patients;1 probably because it reflects the inflammatory severity of COVID-19 added to the chronic inflammation of these patients (previously demonstrated by greater levels of IL-6 and CRP in the blood) [45, 46].

### Mechanical ventilation, gas exchange and extubation

Despite the coexistence of obesity and acute respiratory distress syndrome (ARDS) in our cohort, VT, compliance, and driving pressure did not differ among BMI groups and over time. Obesity-related ventilatory changes include alterations in the respiratory system, drive center, and breathing abnormalities during sleep [47]. Excess abdominal fat displaces the diaphragm upwards, increasing the weight of the chest wall and pleural pressure, thus resulting in decreased residual functional capacity, increased resistance, hypoxemia, and difficult intubation [48]. ARDS is usually characterized by lower compliance, and stiff lungs [49]. Compliance in patients with COVID-19 ARDS seems to be higher in comparison with ARDS of other etiologies, and this may explain why we did not find differences among BMI groups [50]. The optimal parameters of invasive mechanical ventilation in obese patients have not been clearly defined [51]. The recommended tidal volume in obese patients fluctuates between 4–8 ml/kg of predicted body weight and according with the presence of ARDS or not [52]. This is in line with our study in which the volume used was kept within protective ventilatory targets, without differences between the obese and non-obese groups.

In our study, PEEP levels, Plateau pressure, and mechanical power were higher in obese in relation to normal weight and overweight patients. Obese patients require higher levels of PEEP to overcome their restrictive pattern with their typically decreased functional residual, especially in the supine position [53, 54]. The required levels of PEEP in our study were higher in relation to non-obese patients, both at 24, 48 and 72 h. To limit ventilator-induced lung injury (VILI), it has been recommended to maintain plateau pressures below 27 cmH_2_O in obese patients with ARDS, whereas 20 cmH_2_O in patients without ARDS [55–58]. In our study, the plateau pressure differed at 24 and 48 h, being higher in the overweight and obese patients without reaching values above 27 cmH_2_O, suggesting that the PEEP required for these patients did not cause VILI. Driving pressure did not differ between the obese and non-obese patients. Mechanical power refers to the energy transferred from the ventilator to the respiratory system. The proposed limit value to reduce ergotrauma (VILI for mechanical power) is between 17–20 J/min. However, obese patients can tolerate higher values and this population the cut-off point for generating ergotrauma has not been determined [59, 60]. In our study, the mechanical power was higher in obese patients (up to 16 j/min compared to 13 j/min) both at 24 and 48 h. The highest mechanical power values in obese patients correspond to a pathophysiological context because they have higher peak pressures in relation to the increase in airway resistance, especially in the supine position, as well as higher respiratory rate [54, 61].

In our study, we found no differences in gas exchange among BMI groups. Alterations of gas exchange in obese subjects are known as the obesity hypoventilation syndrome, often associated with hypercapnia. This mechanism, at higher altitude is slightly different, since the rate of hypercapnia is lower with a progressive decrease of PaCO_2_ upon ascent, reaching the extremely low value of 8 mmHg at the summit of Mt. Everest [62, 63]. To the best of our knowledge, there are no studies investigating carbon dioxide levels in obese people residing permanently at high altitudes, being highly probable that they do not reach the same values of PaCO_2_ encountered at sea level, as high levels of PaCO_2_ decrease the tolerance to hypoxia [63]. Therefore, lower PaCO_2_ at high altitude could be considered one of the protective factors in COVID-19 [64]. As a confirmation of this, our obese patients had normal PaCO_2_ levels.

Regarding successful extubation, we found significant differences between obese patients and normal patients, being the latter at higher risk of unsuccessful procedure. This is in line with previous studies in which a BMI lower than 28 kg/m^2^ has a higher risk of extubation failure. In contrast, those with obesity or morbid obesity have greater success [65].

### Analytical parameters

We found that at 24 h, ferritin was significantly higher in the overweight group followed by the normal and obesity groups. A cytokine storm is often present in patients with severe COVID-19, and it tends to be associated with a state of hyperinflammation [24, 25, 66]. Obesity is also a condition associated with chronic inflammation. Indeed, obese patients may present an overexpression of pro-inflammatory cytokines such as IL-6 and TNF-α [67]. By adding obesity and COVID-19, a summative effect with an extremely exaggerated immune response can be supposed. Chronic high-altitude exposure can inhibit the immune system, changing the quantity and functionality of immune cells [68–70]. The sympatho-adrenal axis may play a role in the immune adaptations at high altitude [71]. Prolonged exposure to high altitudes may stimulate the innate immune system while suppressing cell-mediated immunity (Th1) [70, 72, 73]. Studies carried out at 2850 m above sea level examined the expression pattern of microRNAs and mRNAs associated with inflammation in T2D monocytes and found that the inflammatory state was reduced even if the patients' lipid profiles were aberrant, and their BMI were higher [74] with less inflammation in obese T2D patients when compared with Dutch T2D patients who live at sea level [75].

### Limitations

Several limitations should be addressed. Our study is monocentric; thus, the findings may be due to correct and well-protocolized management and not to a generality. Moreover, we found some differences with similar evidence published in several studies worldwide and under various geographic and altitude conditions. On the methodological side, this is a small sample, and the design is observational, limiting the value of causal associations. Experimental studies and meta-analyses will be required to strengthen our findings.

## Conclusions

This preliminary study suggests that body mass index was not associated with mortality in critically ill patients at high altitude. Age was associated with an increase in mortality independent of the body mass index. Biomarkers such as ferritin and neutrophils/lymphocytes ratio were independent predictors of mortality in obese patients with COVID-19 at high altitude. These preliminary observations should be further corroborated by larger cohorts and randomized control trials.

## Data Availability

The datasets generated and/or analysed during the current study are not publicly available but are available from the corresponding author on reasonable request.
